# Ten simple rules for writing a technical book

**DOI:** 10.1371/journal.pcbi.1011305

**Published:** 2023-08-10

**Authors:** Jess Haberman, Greg Wilson

**Affiliations:** 1 Anaconda Inc., Austin, Texas, United States of America; 2 Third Bit, Toronto, Ontario, Canada; Dassault Systemes BIOVIA, UNITED STATES

## Introduction

The internet has put a world of technical information just a click away, but that information is often fragmentary, contradictory, incomplete, or out of date. People working in science and technology still need coherent narratives that provide an overview of a subject and lay out a learning path. In other words, they still need books, and whether they are printed or digital, someone has to write them and publish them.

One of us (Haberman) has been helping writers and experts produce instructional content for most of her career: Prior to becoming Director of Learning Solutions at Anaconda, she spent 14 years in book publishing, with several years at O’Reilly Media developing technical books. The other (Wilson) has written four books on programming, coauthored three others, and edited six. The tips below summarize what we’ve learned along the way about writing books that teach people technical subjects.

### Rule 1: Don’t write for money

The first thing any prospective author needs to understand is the economics of publishing. Typically, one-third of the books on a publisher’s list lose money, one-third break even, and one-third are profitable to a greater or lesser extent. For most, it’s “lesser”: Publishers actually make most of their revenue from a handful of bestsellers.

For a technical book, your publisher will typically give you royalties ranging from 10% to 15% of net revenue (i.e., revenue after discounts). You can expect to sell 200 to 2,000 copies; a handful of books do much better than that each year, but only a handful. Downloading has obviously impact this: PDFs and BitTorrent files often appear online within hours of the first digital sales of books (particularly textbooks). Assuming a US$50 price tag, this means that you will probably earn between US$500 and US$7,500. Since it’s going to take at least a thousand hours to write the book, you’ll almost certainly be better off financially doing some consulting (or even getting a part-time job in a fast food restaurant).

You can earn much higher royalties by self-publishing, but doing so comes with many tradeoffs. You won’t have access to the expertise of a publisher when it comes to layout and design, cover art, several levels of editing (development, copyediting, proofreading), printing, print and digital distribution, marketing, and sales. You must dedicate time, money, and energy to finding partners for these tasks or handle them yourself, so choose your path wisely.

So why write a book?

- **To build your reputation**. Many musicians now view albums as a way to get people to buy concert tickets and t-shirts. Similarly, many authors and publishers view books as a way to get people to enrol in tie-in workshops and speaking engagements.
- **Because you enjoy doing it**. Some people like working with wood or wool; you might like working with words.
- **To gain a deeper understanding of the topic**. Writing requires research, and you’ll gain expertise during the writing process.
- **To make the world a slightly better place**. None of us got here without help, and the best way to thank the people who taught us is to teach others.

### Rule 2: Read, then write

Go through two or three of your favorite technical books and make notes about what their authors have done well, such as their use of analogies, how they use diagrams, how much code they show between chunks of prose, and so on. Doing this will give you some ideas for your own book, but it will also help you become more conscious of your writing—in musical terms, it will help you learn how to listen to yourself while you’re playing. One of us (Wilson) learned a lot from the classic Unix books by Kernighan and colleagues [1–4] and from [5], but your tastes may vary.

Reading first will also help you understand what knowledge is already public or well understood, which will help make your arguments even stronger.

### Rule 3: Test your material

Writing a book is a big undertaking, and it’s easy to get discouraged. Instead of tackling it head-on, start blogging regularly (e.g., one article a week, each a few hundred to a thousand words long). Don’t try to write them in the order you think they’ll eventually go into the book: One reason to blog is to get a better idea of what should come before what. As you become more comfortable, though, try writing multipost series that can eventually become chapters.

If you run even a single workshop based on the material for your book while you are writing it, you will almost certainly realize that several of your core assumptions about your audience and material are completely wrong *while you still have time and energy to fix things*. Conferences are always looking for tutorials, your local college or university might be looking for a sessional course, and dozens of learning sites exist to publish training materials (though in this case, you must be careful that their licensing allows you to reuse the material). Your colleagues will probably welcome some lunch and learn sessions, and if all else fails, you can create a YouTube channel to find out how your material sounds.

A corollary is that there’s no point trying material out if you don’t **incorporate what you learn**. If you pay attention to feedback, you will probably rewrite every line of your book at least twice and revise some parts a dozen times or more. You can skip this work if you don’t collect or act on feedback, but your book will almost certainly be a lot worse for it.

We recommend that you drywall and then paint, i.e., that you write a rough draft with placeholders and *then* worry about diagrams, bibliography citations, glossary entries, and so on. For example, the word FIXME appeared over a hundred times in first “complete” draft of [6]; half of those turned out to be irrelevant by the time the text settled down.

An important special case of this is that you should do diagrams on a whiteboard first. It’s faster and more flexible than any computer drawing tool (even fingertip sketching apps for tablets), and a photo taken with a cell phone is good enough for your first readers. Many publishers will re-create your sketches anyway, so don’t fuss over them too much.

#### Union and intersection

No matter how carefully you plan, you will hit part 5 and realize that there was something you should have explained back in part 3. You can fill this in on the fly if you are teaching in person, but that doesn’t work in a book. For this reason, a live lesson should be the *intersection* of what audience members don’t know (i.e., the things you’re sure they all need) while a book has to be the *union* of what readers don’t know (i.e., the things any of them need).

### Rule 4: Start with a learner persona

A *learner persona* is a fictional character that captures key aspects of who you are trying to teach, what they already know, and what they want to learn [6]. Create one or two that are fairly similar (i.e., a primary and secondary audience) and write for them, because a book that’s meant for everyone is actually useful to no one. Imagine what your persona already knows, their career or academic level, and what challenges they face. Your goal is to build on the former to offer solutions to the latter.

As you’re doing this, keep in mind the difference between a *novice* (who is trying to build a mental model), a *competent practitioner* (who has one and wants to fill in gaps in their knowledge), and an *expert* (who wants higher-level discussion of tradeoffs and alternatives). Novices need tutorials that help them build a coherent mental model of the domain; competent practitioners want to fill in the gaps in their knowledge, and experts are usually interested in higher-level discussions of patterns, alternatives, and tradeoffs. No single book can serve all three well.

As a corollary, **don’t write a general introduction to a topic** unless you’re going to be one of the first two or three to market. Instead, focus on a particular aspect (e.g., “Python for Web Scraping”) or a particular audience (e.g., “R for Librarians”). Your potential audience may be smaller, but you’ll reach a much higher percentage of that audience, and a focused book is much easier to write.

And please, **don’t write a reference book**: It can never be as comprehensive or stay as up-to-date as the internet. Tutorials fall out of date too, but if they focus on ideas rather than technical details, they stay relevant for much longer.

### Rule 5: Differentiate yourself

Conduct research to find out what other resources exist for this topic, keeping in mind they don’t have to be books. Where is the audience that you have already identified learning more about the topic right now? What can you offer that differs from and improves on that? The most common weakness in other resources is they are out of date or no longer accurate, but other times, they are poorly organized, not well edited, lack some unique insight you can bring to the topic, or are trying to be too general. Every publisher’s proposal form will ask about competition; finding an answer *before* you have written several chapters can save you a lot of fruitless labor.

Note that some differentiators are not significant enough to be selling points. For example, you might argue that your book is better because it has color diagrams while competing titles are black and white. Color diagrams usually don’t make a significant difference in comprehension, so to an editor, a request for color printing usually equates to more investment and less profit.

Study the style and structure of books you like and mimic a style that works, or tailor the style of your book to match a successful series from your top-choice publisher. The world of publishing is smaller than it was 20 years ago, and the publishers that have survived have focused on replicable series and formats. Whether you want a traditional publisher or you self-publish, don’t re-create the wheel.

### Rule 6: Avoid common mistakes

- **Avoid banal advice** (like “avoid common mistakes”). Sentences like, “You should carefully consider users’ needs,” are frustrating because no sensible person would recommend the opposite. If you can’t provide a checklist of specific things to consider, or a scenario to give the reader insight into how you think through that class of problem, you’re giving them the intellectual equivalent of junk calories.
- **Don’t write for today**. Don’t spend months or years writing a book in such specific detail that a new release of software makes all of your advice obsolete or simply wrong. Instead, generalize and focus on principles that are likely to be long-lived without violating the rule about banality. Your goal should be for your book to remain relevant for 3 to 5 years.
- **Don’t write to cover your ass**. We have read dozens of books that start with a short introduction to XYZ. We cannot remember a single one that was actually useful. If your audience knows Python, they don’t need a chapter-length intro to the language, and if they don’t, one isn’t going to help them. Authors usually include those short introductions so that they can claim the book is suitable for novices when they knew in their hearts it isn’t. Remember, it’s perfectly acceptable to target an audience with more sophisticated knowledge than “none” or “basic.”
- **Don’t copy**. Make sure you credit other people (and AI tools—more on that below) when you use their work or base your work on theirs, especially if you paraphrase or make superficial changes to code examples. Publishers will drop you like a stone if you plagiarize, and telling readers where you got your ideas makes you more credible and helps them figure out what to read next.
- **Don’t try to “write.”** Don’t try to sound like an expert; instead, lean into the idea that you *are* an expert and write in your own voice. You don’t convince readers (or publishers) you have worthwhile opinions by sounding like what you imagine smart people sound like.
- **Don’t try to be funny**. Very few jokes are funny the second time you hear them; even fewer can stand a third retelling unless they’re very, very dry. On a related note, please don’t use exclamation marks—what you’re writing probably isn’t that surprising, and certainly won’t be the second time around—be sparing in your use of bold, italics, and underlining, and remember that nobody reads footnotes.

#### AI writing tools

Seemingly overnight, generative AI tools and large language models (LLMs) have changed the way we write by automating a range of writing tasks. ChatGPT, for example, can help you conquer writer’s block, develop an outline, write an abstract, do competitive research, adjust your tone, or correct your grammar, and the list is only likely to grow. We won’t attempt to predict how these tools will change writing in the long run (which currently means “the next 12 months”); for now, you should treat them the same way you would a spellchecker or grammar checker.

But beware of the pitfalls. AI writing tools frequently hallucinate (i.e., make stuff up), and they do not cite their sources—that’s your job. Before incorporating their output into your work, check your prospective publisher’s stance on them: what do they allow, how do they want your use of these tools acknowledged, and what extra fact-checking do they require for work generated by machines?

### Rule 7: Write a proposal

If your goal is to have your book professionally published, write a proposal before writing the book. You should spend a few weeks on this and make it as complete and convincing as possible; if nothing else, it will help you figure out exactly what you’re writing about.

When you pitch it to your preferred publishers, do some research to find the likely editors so you can be sure they see it instead of it winding up in the slush pile of unsolicited proposals and manuscripts.

#### Shop around

Know what your publishing goals are. Do you prefer the style, brand, or reach of a specific publisher? Are you seeking the top financial offer? Identify your goals before you begin pitching. Don’t feel you have to send your proposal to one publisher at a time, but if you are shopping it around, it is common courtesy to let editors know if the proposal has garnered interest elsewhere. Communicating your goals and timelines can only help you in navigating the pitch process smoothly and achieve your publishing goals.

If you have a strong proposal, it should at the very least land you some time with editors who are knowledgable about the market for the topic. The best editors will help you hone the proposal by asking good questions and steering your book in the right direction.

If you receive an offer for publication, negotiate. Let editors know if you have received or anticipate an offer elsewhere, or if you’re considering self-publishing. There’s no harm in seeking the best offer you can (i.e., the one most aligned with your goals, whatever they are): Even if an offer doesn’t improve, you’ve wasted nothing and can move forward knowing that others are truly invested in your book and will help keep you accountable.

### Rule 8: Automate, but proofread

This is the writer’s equivalent of “trust, but verify.” Depending on the tools you use, you may be able to regenerate all of the examples in your book with a single command, but you must still reread the discussion about those examples every time you make a change to make sure they haven’t fallen out of step. Similarly, it doesn’t matter how well you know your chosen subject: Something will have changed since the last time you looked, and something else will change in the year or more it takes you to complete your manuscript. Your publisher will keep track of errata (i.e., printing errors); it improves longevity of your book if you address them at regular intervals.

Keep in mind that most people won’t read your book linearly: They will skip some sections or jump ahead to find an answer to a problem they need to solve right away. You can help them by making liberal use of cross-references (e.g., “as covered in Chapter 2”) to help them navigate [7]. And because page and chapter numbers frequently change, use chapter titles instead.

Speaking of chapter titles, consider titles that begin with verbs. If you’re writing an instructional book, this will help ensure you’re focused on the task or skill you’re trying to teach. (See [8] for an example.).

### Rule 9: Trust your reviewers and your editor

Lots of people will do cursory reviews or give your manuscript a single careful read; people who will look at changes over and over again and see what’s actually on the page each time are worth their weight in rubies. *Trust them*: If a passage doesn’t make sense to them, then it doesn’t matter if it makes sense to you. Find a colleague or friend who you can trust to be brutally honest. It’s one thing to get feedback from your editor or outside reviewers, but someone you trust who will challenge your arguments is invaluable.

Similarly, as Tom Wilkie once said, an author’s job is to produce the manure in which an editor grows something worth reading. You may sweat over an example for days, but your audience doesn’t see your effort: They see your results. If explanations that seem clear, concise, and elegant to you don’t make sense to your readers, then they’re right and you’re wrong.

A professional editor doesn’t get paid extra for making more edits. So if they mark something, they probably have a good reason. Ask why if you’re not sure. Keep in mind that your motivation and your editor’s are the same, to make the book the best it can possibly be.

Don’t be afraid to set it aside. One of us (Wilson) wrote three quarters of an introduction to R for Python programmers that will probably never see the light of day. This is not unusual: Many authors find that the more they write, the less they believe the book will actually help its intended audience. There is nothing to be ashamed of in this: Scientists don’t expect every hypothesis to turn out to be true, and authors shouldn’t either.

Of course, if you have signed a contract and are suddenly rethinking whether your book should exist, have an honest conversation with your editor about next steps. Don’t hide information, and don’t become impossible to reach. Editors are used to authors becoming discouraged and can provide a much-needed reality check. They can also help you evolve the reasoning, scope, timing, or approach to your book: It’s actually part of their job to do so.

### Rule 10: Stop, ship, and celebrate

Your book will never be perfect. It will probably never even feel *finished*, any more than software does, but you should ship it anyway and then celebrate what you’ve accomplished. Writing a book can be a slog, but it’s not easy to replicate the feeling of accomplishment when you find your book listed online or receive a shipment of printed copies. Don’t be afraid to tell your friends or post on your social media accounts: It isn’t bragging if you actually did it, and if it was worth your time to write the book, it’s worth your time to help people find it.
